# Zibotentan in systemic sclerosis-associated chronic kidney disease: a phase II randomised placebo-controlled trial

**DOI:** 10.1186/s13075-022-02818-6

**Published:** 2022-06-01

**Authors:** Edward P. Stern, Lauren V. Host, Ivy Wanjiku, K. Jane Escott, Peter S. Gilmour, Rachel Ochiel, Robert Unwin, Aine Burns, Voon H. Ong, Helen Cadiou, Aidan G. O’Keeffe, Christopher P. Denton

**Affiliations:** 1 Division of Medicine, University College London, Royal Free Campus, London, UK; 2 Emerging Innovations Unit, BioPharmaceuticals R&D, AstraZeneca, Cambridge, UK; 3 Early Clinical Development, Cardiovascular, Renal & Metabolism, BioPharmaceuticals R&D, AstraZeneca, Cambridge, UK; 4 Joint Research Office, University College London, London, UK; 5 School of Mathematical Sciences, University of Nottingham, Nottingham, UK; 6 UCL Centre for Rheumatology and Connective Tissue Diseases, 2nd Floor – UCL Medical School Building, Royal Free Campus, Rowland Hill Street, London, NW3 2PF UK

**Keywords:** Scleroderma, Renal crisis, Chronic kidney disease, Endothelin, Zibotentan

## Abstract

**Background:**

We report results from a phase II randomised placebo-controlled trial assessing zibotentan, a highly selective endothelin receptor antagonist (ERA), in chronic kidney disease (CKD) secondary to systemic sclerosis (SSc).

**Methods:**

This trial included three sub-studies: ZEBRA 1—a randomised placebo-controlled, double-blind trial of zibotentan in SSc patients with CKD2 or CKD3 (and glomerular filtration rate (GFR) >45 ml/min) over 26 weeks; ZEBRA 2A—a 26-week placebo-controlled, single-blind trial of zibotentan in scleroderma renal crisis patients not requiring dialysis; and ZEBRA 2B—an open label pharmacokinetic study of zibotentan in patients on haemodialysis.

**Results:**

Sixteen patients were screened for ZEBRA 1. Of these, 6 patients were randomised to zibotentan and 7 to placebo. In ZEBRA 1, there were 47 non-serious adverse events (AE) during the trial. Twenty-seven occurred in the placebo group and 20 in the zibotentan group. One serious adverse event (SAE) occurred during ZEBRA1, in the placebo arm. Descriptive statistics did not suggest an effect of study drug on serum sVCAM1. Estimated GFR numerically declined in patients treated with placebo at 26 weeks and 52 weeks. In contrast, average eGFR increased in zibotentan-treated cases.

The 4 patients in ZEBRA 2A experienced 8 non-serious AEs, distributed equally between placebo and zibotentan. There was one SAE each in placebo and zibotentan groups, both unrelated to study medication.

ZEBRA 2B recruited 8 patients, 6 completed first dosing, and 2 completed a second dosing visit. Pharmacokinetic analysis confirmed zibotentan levels within the therapeutic range. Three patients experienced 3 non-serious AEs. One SAE occurred and was unrelated to study drug.

**Conclusions:**

Zibotentan was generally well-tolerated. ZEBRA 1 did not show any effect of zibotentan on serum sVCAM-1 but was associated with numerical improvement in eGFR at 26 weeks that was more marked at 52 weeks. ZEBRA 2B suggested a feasible dose regimen for haemodialysis patients.

**Trial registration:**

EudraCT no: 2013-003200-39 (first posted January 28, 2014)

ClinicalTrials.gov Identifier: NCT02047708

Sponsor protocol number: 13/0077

## Background

Scleroderma (systemic sclerosis; SSc) is a multisystem rheumatic disease that results in vascular damage and fibrosis of target organs [1]. Although uncommon, affecting around 1 in 10,000 of the UK population, SSc is important by virtue of its high mortality (more than half of patients diagnosed with SSc eventually die from the disease) [2] and a large non-lethal morbidity.

Renal involvement is important in SSc [3]. Historically, there has been a focus on scleroderma renal crisis (SRC) which most often develops in early stage diffuse cutaneous SSc [4] and is strongly associated with certain clinical and serological features including a diffuse skin subset, worsening of skin severity, tendon friction rubs, and presence of antinuclear autoantibodies targeting RNA polymerase III antigens [5]. Recent work has highlighted the potential role of genetic polymorphism and perturbed Wnt signalling as additional susceptibility factors within anti-RNA polymerase antibody positive patients [6]. Although once almost always fatal, most patients now survive the acute crisis due to routine use of ACE inhibitors [7]. However, SRC can lead to chronic kidney disease (CKD). In addition, CKD can occur in SSc without SRC, and this may reflect multiple mechanisms including fibrosis, vasculopathy, and overlap connective tissue disease (CTD) as well as common mechanisms not directly related to SSc [8]. A recent study of 5 pooled cohorts reported that around one fifth of SSc patients had eGFR of less than 60ml/min [9]. CKD has been shown to predict poor outcome in SSc [10, 11].

Endothelin is implicated as a pathogenic driver of vasculopathy and other manifestations of SSc, acting via two high affinity G-protein coupled receptors ETRA and ETRB [12]. Targeting the endothelin axis with non-specific receptor antagonists such as bosentan and macitentan has proven beneficial in SSc for pulmonary hypertension [13] and for digital vasculopathy [14]. In addition, a small open-label study (BIRD-1) suggested potential benefit for CKD following SRC with greater recovery of renal function in cases treated with bosentan [15], although another study failed to show improved short-term outcome for SRC [16]. Endothelin receptor antagonists can be classified as non-selective, such as bosentan or macitentan, or selective, depending on their relative affinity for the endothelin receptor A and B subtypes, and this may potentially influence efficacy and tolerability [12]. In the present study, we describe the results of a study evaluating zibotentan, a highly selective endothelin A receptor antagonist [17], in systemic sclerosis patients with renal involvement, focusing on CKD.

Building on previous work supporting the rationale and feasibility of a non-selective endothelin receptor antagonist use in SSc-associated CKD after SRC [14], the hypothesis of this study is that chronic treatment with a highly selective endothelin A receptor antagonist will have a beneficial effect on laboratory and clinical manifestations in patients with SSc-CKD [8].

Here we report the results of a phase II, single-centre, randomised placebo-controlled, 3-part trial to assess the safety, tolerability, and efficacy of zibotentan in patients with renal disease secondary to scleroderma. Study objectives were, first, to assess the tolerability, safety, and effect of zibotentan treatment over 6 months on renal biomarkers (e.g. serum VCAM1) [18] in patients with scleroderma associated with CKD2 and CKD3 (eGFR>45 ml/min/1.73 m^2^) (ZEBRA1); second, to assess the tolerability, safety, and effect of zibotentan treatment over 6 months on renal function (GFR) in patients who have experienced a Scleroderma Renal crisis (SRC) not requiring dialysis (ZEBRA2A); and third, to evaluate the effect of end-stage kidney disease (ESKD) and haemodialysis on the tolerability, safety, and pharmacokinetic profile of a single dose of zibotentan in patients with severe CKD (ZEBRA 2B).

Overall, this project advances our understanding of the role of the endothelin axis in the renal pathology of SSc and provides a platform to test emerging biomarkers of renal disease in SSc.

## Methods

### Study design

This was a phase II, single-centre, randomised, placebo-controlled, three-part trial to assess the safety, tolerability, and efficacy of zibotentan in patients with renal disease secondary to scleroderma. The trial included three sub-studies:

- ZEBRA 1 was a 1:1 randomised parallel group placebo-controlled, double-blind, single-centre trial comparing zibotentan 10 mg once daily orally, as used in previous clinical trials (with possible dose reductions to a minimum dose of 5 mg once daily in the event of side effects such as fluid retention) with matched placebo in SSc patients with chronic kidney disease (CKD) CKD2 and CKD3A reflecting the current KDIGO (Kidney Disease: Improving Global Outcomes) clinical practice guidelines [19] and requiring an estimated glomerular filtration rate (eGFR) >45 ml/min/1.73 m^2^) over 26 weeks.
- ZEBRA 2A was a parallel group placebo-controlled, single-blind, trial comparing zibotentan once daily orally over 26 weeks, with a 26-week follow-up, with matched placebo using 2:1 (active: placebo) randomisation, in patients within 1–12 months of SRC not requiring ongoing renal replacement therapy. Individual patients were started at 2.5 mg once daily and following weekly monitoring were dose escalated by 2.5 mg weekly to a maximum of 10 mg once daily over the course of the first 4 weeks.
- ZEBRA 2B was an open-label single ascending dose administration pharmacokinetic (PK) study of zibotentan 2.5 mg to 10 mg orally in patients requiring dialysis for end stage kidney disease (ESKD). Individual patients received up to two single doses of zibotentan (at different dose levels).
- The study protocol was approved by the NHS ethics committee (IRAS number: 136274), and all patients gave written informed consent in accordance with the International Council for Harmonisation of Technical Requirements for Pharmaceuticals for Human Use (ICH) and Good Clinical Practice (GCP) before any study procedures were performed.

### Participants

Eligible participants were >18 years of age with either diffuse or limited systemic sclerosis of any disease duration (except for ZEBRA 2B, see below). Females of childbearing potential and males were required to use one highly effective and one other method of contraception from the time of consent until 6 weeks after treatment discontinuation.

For ZEBRA 1 patients were required to have CKD2 or CKD3A, i.e. eGFR>45 ml/min/1.73 m^2^. People with an eGFR of 60–89 ml/min/1.73 m^2^ (inclusive) without any evidence of kidney damage or disease were not considered to have CKD2. They could not have had SRC within the previous 12 months.

For ZEBRA 2A, patients had to have experienced SRC within 1–12 months of study start date defined by new onset of blood pressure >150/85 mmHg obtained at least twice over a consecutive 24-h period AND decline in renal function as defined by an increase of at least 10% from a baseline serum creatinine measured within the previous 12 months. SRC was corroborated by at least one of the following: microangiopathic haemolytic anaemia on blood smear, retinopathy typical of acute hypertensive crisis, new onset of urinary red blood cells (excluding other causes), flash pulmonary oedema, oliguria or anuria, and renal biopsy findings consistent with SRC. These determinants align with recently proposed international SRC classification criteria [20]. There was no pre-specified eGFR cut-off, but patients were not eligible if requiring renal replacement therapy.

Diagnosis of SSc was not required for ZEBRA 2B which was a pharmacokinetic study in patients receiving regular haemodialysis treatment for ESKD from any cause. Patients were not eligible for any substudy if they had received an endothelin receptor antagonist within the previous 3 months. Other medications for SSc or concomitant medical conditions were permitted.

ZEBRA 1 was randomised in a 1:1 allocation, ZEBRA 2A was randomised 2:1, active:placebo, and was a single blind trial, so the specific treatment assigned was provided for the investigators, allowing dose titration in response to plasma drug concentration.

For ZEBRA 1 and ZEBRA 2A, following screening, there were 12 study visits at baseline, week 1 and every 4 weeks to week 24, then at weeks 26, 27, and 30. There was a final safety visit at week 52. For ZEBRA 2B following screening, there was one treatment visit with pharmacokinetic (PK) sampling and a second visit at 1 week after dosing.

### Endpoints

For ZEBRA 1, the primary endpoint was safety and tolerability, assessed by adverse events (AEs) and serious AEs (SAEs) and change from baseline to 26 weeks in serum VCAM-1, a candidate biomarker of SSc renal involvement [16]. For ZEBRA 2A, the primary endpoint was safety and tolerability assessed AEs and SAEs and change in eGFR from baseline to week 26. Previous studies have confirmed the reliability of calculated eGFR in SSc [18]. For ZEBRA 2B, the primary endpoints were number of AEs, the number and nature of SAEs, and zibotentan plasma concentrations (ng/ml) recorded at the following times (in hours) since dosage: 0 (baseline), 3, 24, and 30 h.

When not considered primary endpoints, other secondary outcomes for ZEBRA 1 and ZEBRA 2A were eGFR, serum and urine ET-1, MCP-1, sVCAM-1, and sICAM-1. All urine analytes were expressed as urinary analyte to creatinine ratio to correct for differences in urinary volume for each patient sample [8].

The following samples were processed centrally by routine clinical analyser methods at The Royal Free London NHS Foundation Trust laboratories: haematology, clinical chemistry, and urinalysis. eGFR was calculated using the abbreviated MDRD equation. A bedside dip stick test was used for the analysis of urine protein, blood, and glucose. Biomarker analysis for the formal trial endpoints (sVCAM-1, VEGF, vWF, ET-1, sICAM-1, MCP-1) was conducted using commercial assay kits (R&D Systems, Abingdon, Oxford, UK) in the research laboratories of the Centre for Rheumatology. PK plasma samples were analysed using LC-MS/MS by York Bioanalytical Solutions Ltd.

### Statistical analysis

Sample size for ZEBRA was based upon expected frequency of CKD in a large single-centre SSc cohort and previous studies examining candidate biomarkers in SRC, together with expected variability in endpoints and potential treatment effect of an ERA on eGFR [15]. The target recruitment was 48 patients for ZEBRA 1 and 12 patients with SRC not requiring dialysis for ZEBRA 2A. For ZEBRA 2B, it was anticipated that up to 12 patients with renal failure of any cause would be recruited from the regular dialysis programme at the Royal Free Hospital.

For ZEBRA 1, the number of adverse events and the number and nature of serious adverse events, stratified by group (placebo and active treatment) are reported and whether a SAE is related to the investigational medicinal product. Serum sVCAM-1 levels are summarised by treatment group at time 2 (week 26) for patients in ZEBRA 1. The summary included mean, median, standard deviation, and maximum and minimum values. Values at 52 weeks are also presented according to previous treatment allocation.

For ZEBRA 2A, the number of adverse events and the number and nature of serious adverse events, stratified by group (placebo and active treatment), are reported. In addition, whether an SAE was related to the investigational medicinal product was reported. The eGFR level is reported directly and stratified by the treatment group at time 2 (week 26) for patients in ZEBRA 2A, owing to the small number of patients who were recruited to ZEBRA 2A.

For ZEBRA 2B, the number of adverse events and the number and nature of serious adverse events are reported. In addition, whether an adverse event is related to the investigational medicinal product is reported. For each patient in ZEBRA 2B, the zibotentan blood plasma concentration is plotted against the time since dosage (with concentration recorded at approximately 3, 24, and 30 h post-dose). The 24- and 30-h samples are pre- and post-dialysis respectively.

Each secondary outcome is summarised by the treatment group at each time point. For ZEBRA 1, the summary includes mean, median, standard deviation, and maximum and minimum values. For ZEBRA 2A, a direct report of each secondary outcome is made at each time point, owing to the small number of patients recruited to this sub-study.

## Results

### Patient disposition and baseline characteristics

For ZEBRA 1 and ZEBRA 2A, target recruitment was not achieved. This limits substantially the statistical power and interpretation of the trial results for both sub-studies. Under-recruitment was a consequence of the lower frequency of clinically significant CKD in the Royal Free SSc patient cohort at the time of enrolment [9], and several challenging issues relating to study medication manufacture and database management that resulted in a shorter than anticipated recruitment period. Despite the substantial operational challenges encountered, patients were enrolled into all studies, and this represents the first randomised placebo-controlled trial directly examining treatment of renal disease in SSc. For the ZEBRA 2B sub-study a diagnosis of SSc was not required, and this enabled appropriate recruitment and dosing that was within the target number of doses to be examined and yielded valuable information on pharmacokinetics in patients on long-term haemodialysis.

**Fig. 1:**
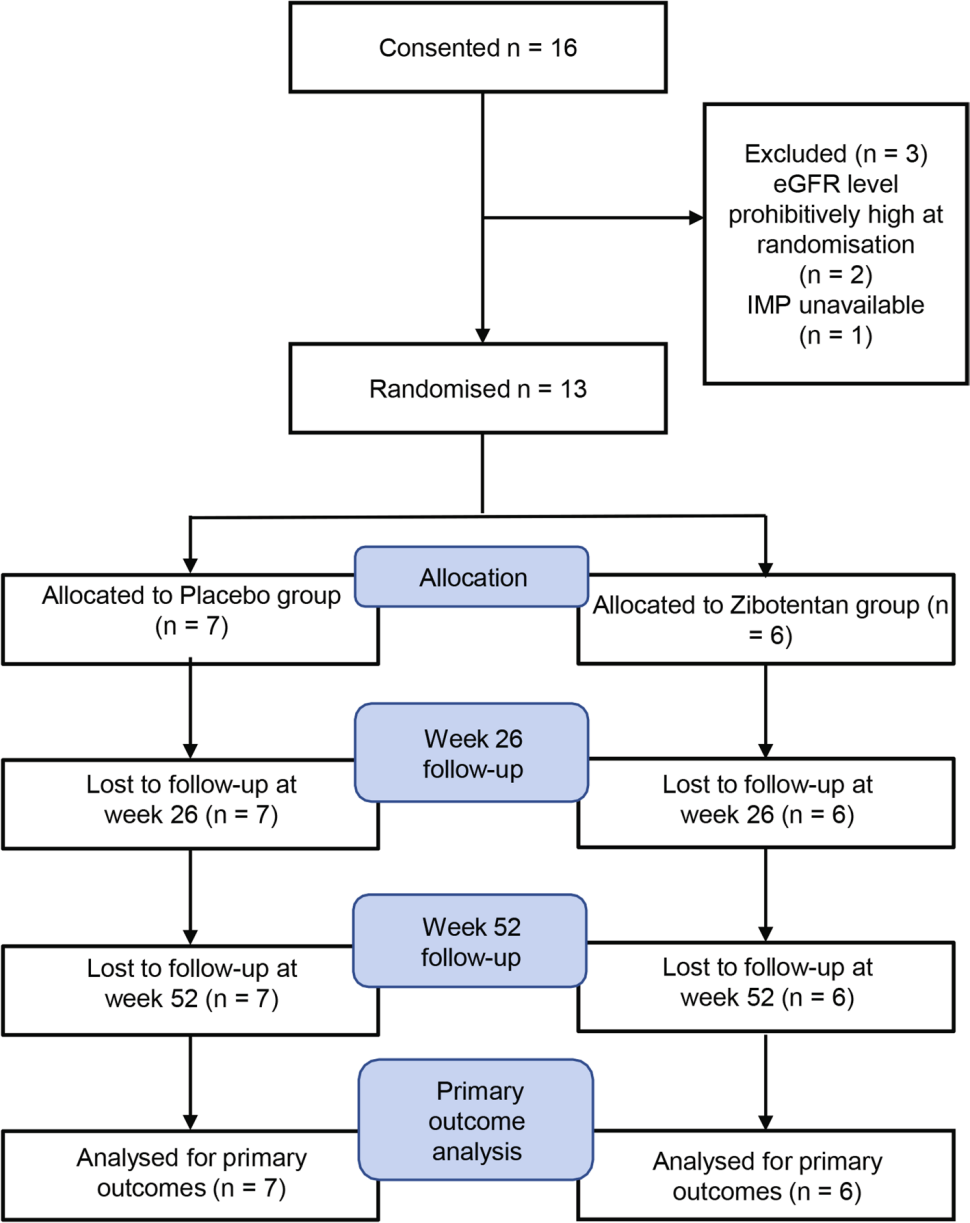
CONSORT diagram showing flow of patients through the ZEBRA 1 sub-study. The diagram provides a summary of the flow of patients through the ZEBRA 1 sub-study including screened cases and 13 patients randomised

Figure 1 shows a CONSORT diagram which provides a summary of the flow of patients through the ZEBRA 1 sub-study. Of the 16 consenting patients, 1 failed screening and 1 was excluded due to IMP availability. A total of 13 patients were recruited to take part in the ZEBRA 1 sub-study. Of these, 6 patients were randomised to receive zibotentan (up to 10 mg once daily orally and henceforth known as the ‘zibotentan group’) and 7 patients were randomised to receive a placebo (henceforth known as the ‘placebo group’). The demographic and clinical features of the study cohort are summarised in Table 1 showing a generally good balance between the treatment arms, including relevant concomitant medication, such as vasodilator treatment or disease-modifying drugs. The cause of CKD for these patients was likely to be multifactorial but in 1 case there was a previous history of SRC.

**Table 1:** Summary of demographic and clinical characteristics of ZEBRA trial study cohort

**ZEBRA 1**		**Placebo**	**Zibotentan**
*n*=		7	6
Sex	Male	1	2
	Female	6	4
Ethnicity	White	7	6
Subset	Diffuse	2	1
	Limited	5	5
ANA			
	ACA	5	3
	ARA	1	2
	ATA	0	0
	Other	1	1
Age (years) mean (SD)		65 (7)	62 (7)
SSc duration (years) mean (SD)		9 (7)	11 (7)
SRC, *n* (%)		1 (14%)	0
PH, *n* (%)		0	0
DU, *n* (%)		2 (28%)	2 (33%)
Vasodilator, *n* (%)		5 (71%)	5 (83%)
DMARD, *n* (%)		1 (14%)	1 (17%)
**ZEBRA 2A**		**Placebo**	**Zibotentan**
*n*=		2	2
Sex	Male	0	1
	Female	2	1
Ethnicity	White	2	1
	Asian	0	1
Age (years)		57, 34	63, 65
SSc duration (years)		1, 2	1, 1
SRC (*n*)		2	2
PH (*n*)		0	0
DU (*n*)		0	0
Vasodilator (*n*)		2	2
DMARD (*n*)		1	2
**ZEBRA 2B**			
*n*=			6
Sex	Male	NA	5
	Female	NA	1
Ethnicity	White	NA	4
	Black	NA	1
	Mixed-race		1

*SRC* Scleroderma renal crisis, *PH* Pulmonary hypertension, *DU* Digital ulceration, *DMARD* Disease-modifying therapy (methotrexate or mycophenolate mofetil)

Due to recruitment difficulties, only four patients were enrolled into ZEBRA 2A. Of these, two were randomised to receive zibotentan (henceforth known as the ‘zibotentan group’) and two were randomised to receive a placebo (henceforth known as the ‘placebo group’). One patient, who was allocated to the ‘zibotentan group’ (Z2A004) withdrew from the trial during the second week of follow-up because this patient was admitted to hospital with community-acquired pneumonia and longer-term outcome data were not available for this patient.

A total of 8 patients were recruited to take part in the ZEBRA 2B open label sub-study. This sub-study did not include randomisation, but it was planned that each recruited patient would receive a 2.5 mg dose of zibotentan at their first visit followed by 5.0 mg of zibotentan at their second visit. Of the 8 patients recruited, 6 completed a first dosing visit and 2 completed a second dosing visit. The two recruited patients who did not complete a first (or second) visit failed screening following recruitment and were not eligible to receive a dose of zibotentan.

Four recruited ZEBRA 2B patients completed a first dosing visit, but not a second dosing visit because three patients declined to receive a second dose of zibotentan, and one patient scheduled to receive a second dose of zibotentan missed the clinic visit.

### Safety analysis for ZEBRA 1, ZEBRA 2A, and ZEBRA 2B

Primary analysis for safety and tolerability showed that patients randomised in ZEBRA 1 experienced a total of 47 non-serious adverse events during the trial. Of these non-serious adverse events, 27 occurred amongst patients in the placebo group and 20 occurred amongst patients in the zibotentan group. Six patients in the placebo group experienced at least one non-serious adverse event. Five patients in the zibotentan group experienced at least one non-serious adverse event. Fluid retention leading to lower limb oedema occurred as an adverse event in 2 patients on zibotentan, and none on placebo. In both cases, ankle swelling developed within 1 month of first dosing and was judged as possibly or probably related to study medication. One patient continued treatment, but in the other case study drug was discontinued after 8 weeks and ankle swelling subsequently resolved. Minor weight gain within the first 4 weeks of commencing study medication, possibly reflecting fluid retention, was more frequent in zibotentan than placebo arms, but was not considered clinically significant (data on file). There was one serious adverse event during the trial. This was in the placebo group and was *Streptococcus pneumoniae* pneumonia that was graded as severe, but unrelated to study medication. Although generally safe and well tolerated, there was a clear imbalance in AEs related to fluid retention in the active treatment arm of ZEBRA 1 that meant that some patients needed dose reduction during the study, suggesting 5mg may be a more acceptable dose in any future studies.

In ZEBRA 2A, the patients experienced 8 non-serious adverse events during the trial. Of these non-serious adverse events, four occurred amongst patients in the placebo group and four occurred amongst patients in the zibotentan group. All patients in both the placebo group and the zibotentan group experienced at least one adverse event. Patients recruited to ZEBRA 2A experienced two serious adverse events during the trial. One was a pericardial effusion occurring in the placebo group and graded as moderate severity and the other, a community acquired pneumonia graded as severe occurred in the zibotentan group. Neither SAE was considered related to study medication.

Three patients recruited to ZEBRA 2B experienced a total of three non-serious adverse events during the trial. There was one serious adverse event in ZEBRA 2B, a pseudoaneurysm of arteriovenous fistula (AVF) and raised international normalised ratio (INR) occurring 7 weeks after single dose of zibotentan, graded moderate severity and unrelated to study drug.

### Efficacy analysis for ZEBRA 1 and ZEBRA 2A

**Fig. 2:**
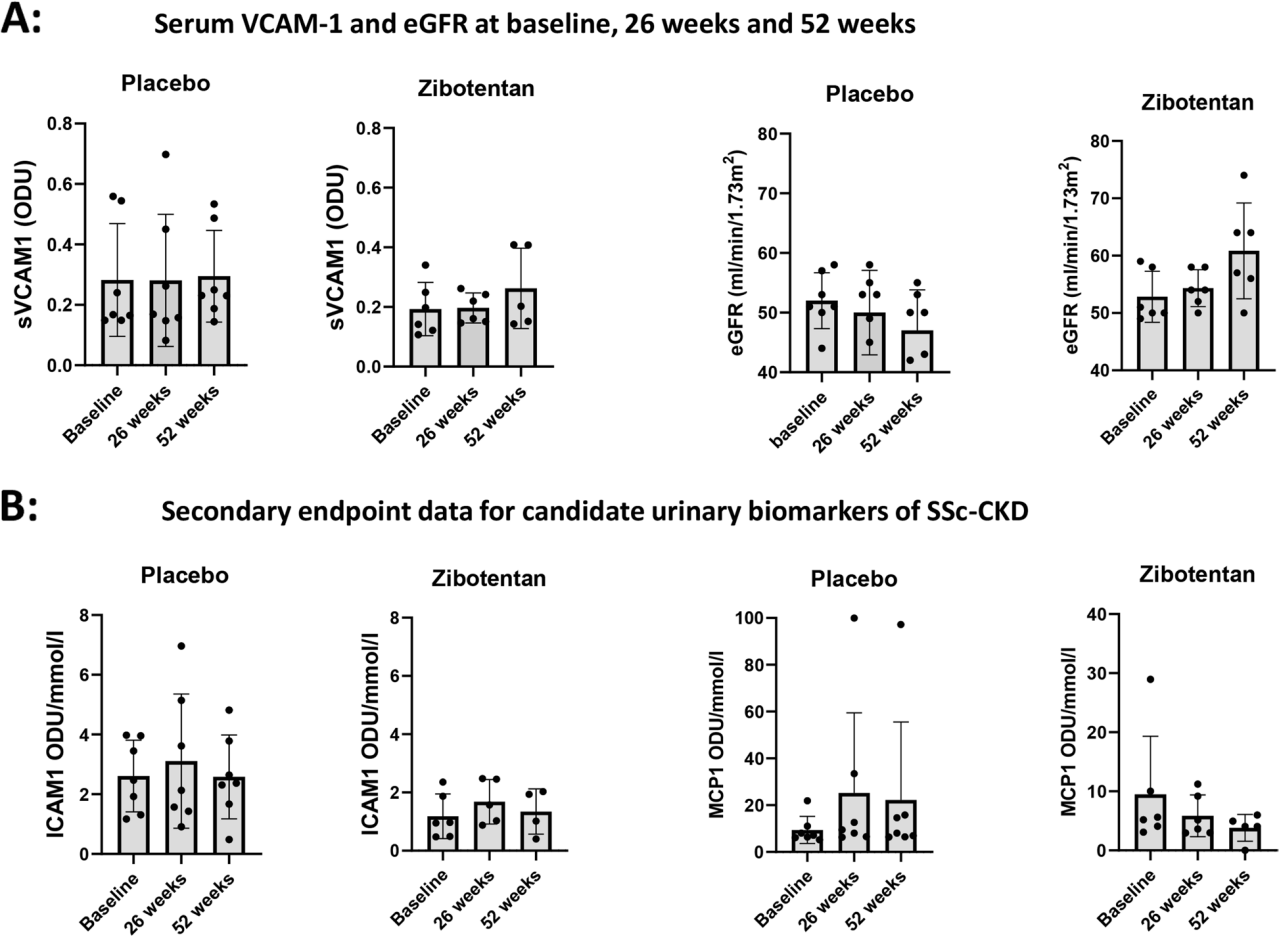
Representative primary and secondary endpoint data for ZEBRA 1 sub-study. **A** Endpoint data for serum VCAM-1 and eGFR in ZEBRA 1. Upper panels show the serum VCAM-1 levels at baseline, 26 weeks, and 52 weeks for the patients in placebo and zibotentan groups. Mean and SD are indicated. There was no apparent difference between treatment groups. The lower panel shows that eGFR (ml/min/1.73m2) decreased in the placebo arm and increased in the zibotentan arm at 52 weeks. **B** Candidate CKD-SSc urinary biomarker data for ZEBRA 1. Secondary endpoint data for candidate urinary biomarkers of SSc-CKD identified in a previous cohort study [8]. For urinary ICAM-1 to creatinine ratio, there is no difference between treatment groups or timepoints. For MCP-1 to creatinine ratio, the placebo group shows increasing level at 52 weeks compared to a numerical reduction for the zibotentan group although distribution of data is wide as shown by SD for each time point

Data for primary and secondary efficacy analysis in ZEBRA 1 are described in Table 2. The primary efficacy outcome was serum sVCAM-1 based on previous data suggesting that this may be a marker of SRC, a form of acute kidney injury [16]. However, in ZEBRA 1, which represents a cohort of CKD, there was no effect of zibotentan on circulating levels of sVCAM-1 between baseline and 26 weeks or at 52 weeks. There was a wide range of values at baseline between the two treatment arms as shown in Fig. 2A and this may have limited interpretation of findings. However, based on more recently published findings from our group [8] that were not available when the trial was designed, we do not now consider serum sVCAM-1 to be a useful or interpretable marker of SSc-CKD or one that may be influenced by zibotentan treatment.

**Table 2:** Endpoint data for ZEBRA 1 trial at baseline, 26 weeks, and 52 weeks

Variable	Group	***n***	Mean	Std. Dev.	Median	Min.	Max.
Serum VCAM-1 Level at baseline (ODU)	Placebo	7	0.28	0.19	0.17	0.15	0.56
	Zibotentan	6	0.19	0.09	0.17	0.11	0.34
Serum VCAM-1 Level at week 26 (ODU)	Placebo	7	0.28	0.22	0.17	0.08	0.7
	Zibotentan	6	0.2	0.05	0.19	0.15	0.26
Serum VCAM-1 Level at week 52 (ODU)	Placebo	7	0.29	0.15	0.23	0.14	0.53
	Zibotentan	5	0.26	0.13	0.2	0.14	0.41
eGFR at baseline (ml/min/1.73m2)	Placebo	7	52	4.69	51	44	58
	Zibotentan	6	52.83	4.45	50.5	49	59
eGFR at week 26 (ml/min/1.73m2)	Placebo	7	50	7.09	53	37	58
	Zibotentan	6	54.33	3.20	54	50	58
eGFR at week 52 (ml/min/1.73m2)	Placebo	7	47	6.83	50	36	55
	Zibotentan	6	60.83	8.35	60.5	50	74
Serum ET-1 level at baseline (ODU)	Placebo	7	0.2	0.08	0.17	0.15	0.38
	Zibotentan	6	0.19	0.06	0.18	0.11	0.3
Serum ET-1 level at week 26 (ODU)	Placebo	7	0.19	0.09	0.16	0.1	0.4
	Zibotentan	6	0.18	0.04	0.18	0.13	0.24
Serum ET-1 level at week 52 (ODU)	Placebo	7	0.17	0.07	0.15	0.11	0.33
	Zibotentan	5	0.24	0.04	0.22	0.21	0.3
Serum MCP-1 level at baseline (ODU)	Placebo	7	0.24	0.12	0.2	0.11	0.48
	Zibotentan	6	0.22	0.07	0.21	0.13	0.32
Serum MCP-1 level at week 26 (ODU)	Placebo	7	0.27	0.17	0.23	0.11	0.59
	Zibotentan	6	0.17	0.04	0.15	0.14	0.25
Serum MCP-1 level at week 52 (ODU)	Placebo	7	0.23	0.09	0.22	0.12	0.4
	Zibotentan	5	0.29	0.19	0.16	0.13	0.57
Serum ICAM-1 level at baseline (ODU)	Placebo	7	0.67	0.22	0.7	0.31	1.03
	Zibotentan	6	0.74	0.16	0.7	0.58	0.98
Serum ICAM-1 level at week 26 (ODU)	Placebo	7	0.68	0.33	0.66	0.14	1.21
	Zibotentan	6	0.77	0.14	0.79	0.56	0.91
Serum ICAM-1 level at week 52 (ODU)	Placebo	7	0.74	0.3	0.74	0.37	1.22
	Zibotentan	5	0.83	0.17	0.92	0.56	0.98
Urine MCP-1:creatinine	Placebo	7	9.4	5.82	7.1	5.23	21.9
ratio at baseline (ODU/mmol/l)	Zibotentan	6	9.49	9.82	5.41	3.09	28.94
Urine MCP-1:creatinine ratio at week 26 (ODU/mmol/l)	Placebo	7	25.21	34.29	9.49	6.33	99.92
	Zibotentan	6	5.85	3.52	4.37	2.94	11.21
Urine MCP-1:creatinine	Placebo	7	22.21	33.32	8.08	6.39	97.24
ratio at week 52 (ODU/mmol/l)	Zibotentan	4	4.77	0.91	4.51	4.06	5.98
Urine ICAM-1:creatinine ratio at baseline (ODU/mmol/l)	Placebo	7	2.61	1.2	2.47	1.17	3.98
	Zibotentan	6	1.18	0.76	0.96	0.47	2.35
Urine ICAM-1:creatinine ratio at week 26 (ODU/mmol/l)	Placebo	7	3.11	2.24	2.13	0.91	6.96
	Zibotentan	6	10.83	22.43	2.01	0.88	56.6
Urine ICAM- 1:creatinine ratio at	Placebo	7	2.58	1.4	2.37	0.48	4.82
week 52 (ODU/mmol/l)	Zibotentan	4	1.35	0.78	1.48	0.4	2.03
Urine VCAM-1:creatinine ratio at baseline (ODU/mmol/l)	Placebo	7	85.73	207.91	3.45	0.36	556.61
	Zibotentan	6	2.63	4.31	0.88	0.71	11.43
Urine VCAM- 1:creatinine ratio at	Placebo	7	58.53	131.85	5.51	0.33	356.79
week 26 (ODU/mmol/l)	Zibotentan	6	10.76	22.04	2.11	0.09	55.64
Urine VCAM-1:creatinine ratio at week 52 (ODU/mmol/l)	Placebo	7	98.87	237.13	2.65	0	635.95
	Zibotentan	4	1.58	0.44	1.41	1.29	2.23

Although analysis and interpretation of renal function and protein analyte levels in serum and urine are limited by the small number of patients recruited into ZEBRA 1, there were some notable findings amongst some of the secondary efficacy end points that could be explored in future studies. Thus, whilst eGFR (ml/min/1.73 m^2^) was well matched at baseline with mean (SD) of 52.0 (4.7) for placebo and 52.8 (4.5) for zibotentan groups, there was a trend for improvement in eGFR at 26 weeks in the zibotentan group (54.3 (3.2)) that was more marked at 52 weeks (60.8 (8.4)) compared with slight reduction in mean eGFR for the placebo group to 50 (7.1) and 47 (6.8) at 26 and 52 weeks respectively. This is notable as it may suggest beneficial impact on renal function that continues after the cessation of treatment and so is unlikely to simply reflect acute haemodynamic effects of zibotentan, but rather supports longer term disease modification.

All analysis of ZEBRA 1 candidate biomarker data in serum and urine must be circumspect due to small sample size. In this context, no major differences between potential treatment effect were observed at week 26 compared with baseline. Of many candidate markers examined in serum and urine as secondary endpoints in this trial, urinary MCP-1 and urinary sICAM-1 are perhaps the most interesting because they have recently been identified as candidate markers of SSc-CKD in other studies [8]. These two urinary markers are shown in Fig. 2B. For urinary sICAM-1, there is no apparent difference between treatment arms but urinary MCP-1: creatinine ratio levels declined on zibotentan and increased in placebo treated patients. Thus, baseline (mean (SD) ODU/mmol/L) urinary MCP1/creatinine was 9.4 (5.8) in placebo arm and 9.5 (9.8) in active treatment arm, rising to 25.2 (34.3) at 26 weeks on placebo and falling to 5.9 (3.2) on zibotentan. These trends remained different at 52 weeks at 22.2 (33.3) in the placebo-treated arm and 4.8 (0.9) for the zibotentan-treated group.

It was not possible to undertake any meaningful efficacy analysis for ZEBRA 2A due to the small number of patients recruited. However, there was no change in eGFR at week 26 for the single evaluable zibotentan treated patient. At 52 weeks, all three evaluable patients, including 2 treated with placebo, showed numerical improvement in eGFR. Data for serum and urinary analytes are included in Table 2 but are insufficient for interpretation since only a single treated patient was available for analysis.

### Endothelin concentrations in patients receiving zibotentan

As shown in Table 2, the levels of circulating endothelin 1 in serum measured by ELISA were similar at baseline between the two treatment arms. In contrast to previous findings using the non-selective ERA bosentan that led to marked increase in circulating endothelin 1 levels [12], there was no significant increase in endothelin levels at week 26 suggesting that in the absence of ETRB blockade, an important scavenger receptor for plasma endothelin in the lung [21], there is not an increase in circulating endothelin 1 during treatment that may be associated with rebound hypertension at discontinuation of study drug. There were no instances of this occurring as an adverse event after week 26 of the trial.

### Zibotentan blood plasma concentration

**Fig. 3:**
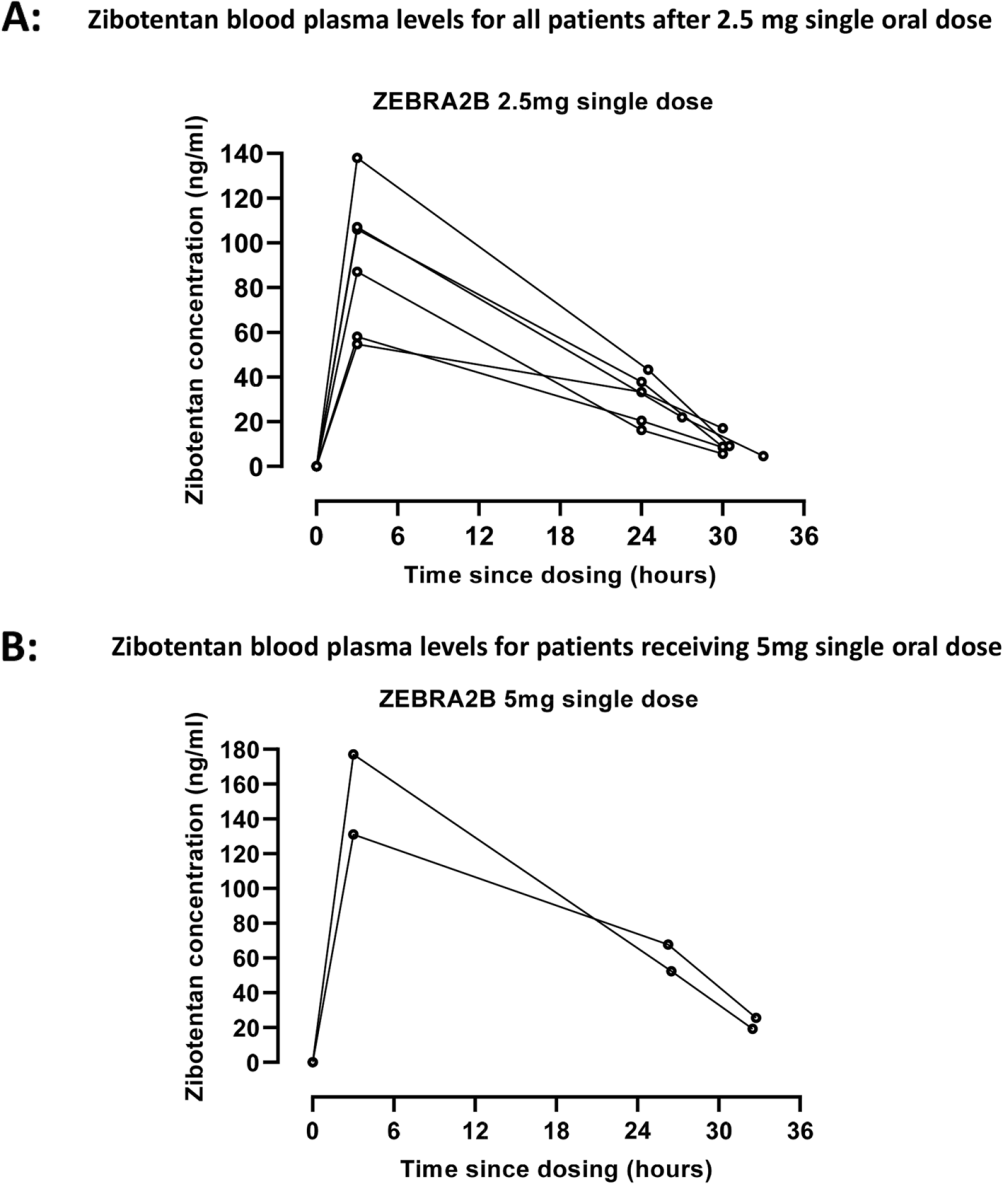
Pharmacokinetic data following single oral dose zibotentan for patients in ZEBRA 2B. **A** Zibotentan blood plasma levels for all patients after 2.5 mg single oral dose. The upper panel shows the concentration of zibotentan (ng/ml) at 6 after a single oral dose of 2.5 mg zibotentan in six patients on haemodialysis. The final samples were taken pre- and post-dialysis to explore clearance of zibotentan. **B** Zibotentan blood plasma levels for patients receiving 5 mg single oral dose. Concentration of zibotentan (ng/ml) for the two patients that were re-dosed with 5 mg oral zibotentan is plotted against time with the final two samples being pre- and post-haemodialysis

Pharmacokinetic analysis was undertaken in ZEBRA 2B for all patients who received doses of the IMP. Blood plasma concentrations of zibotentan were measured at each time point post-dosage, for each dose received. The measurement schedule was designed to take place at approximately 3, 24, and 30 h post-dose. Twenty-four- and 30-h samples were pre- and post-haemodialysis respectively. Blood plasma concentration over time is shown for all six patients receiving 2.5 mg (Fig. 3A) and individually for patients later receiving a 5 mg dose (Fig. 3B). Overall, the results of this study confirmed feasibility of administration in patients on dialysis and indicated potential dosing to achieve expected plasma concentration.

## Discussion

The present ZEBRA phase II trial has explored the feasibility and safety of using a highly selective endothelin receptor antagonist, zibotentan, in patients with chronic kidney disease complicating systemic sclerosis. The background to this study is the established efficacy of selective and non-selective ERA in management of other major complications of SSc including pulmonary arterial hypertension [10]. In addition, bosentan is approved for management of digital ulcer disease [11]. In an earlier study, our group provided the rationale for targeting the endothelin axis in scleroderma renal disease and reported a small open label study of bosentan in patients that had suffered scleroderma renal crisis (SRC). This open-label study suggested that patients with residual renal impairment after SRC may show greater recovery of renal function as assessed by eGFR over 12 months following 6 months treatment with bosentan [14].

Zibotentan was generally well tolerated with no imbalance in serious adverse events between treatment arms or any evidence of drug related SAE. However, AEs were seen in most patients and included fluid retention and weight gain that required medical management and on occasion led to treatment discontinuation. This suggests that fluid retention is likely to be due in part to selective ETRA antagonism and is in line with previous trials evaluating zibotentan [22].

As a highly selective ERA, it was considered that zibotentan could have advantages in treating SSc-CKD over a non-selective ERA such as bosentan, because the endothelin B receptor subtype has been implicated in vasodilation and in clearance of circulating endothelin that may in part explain the elevation of endothelin levels seen in patients receiving bosentan and perhaps underlie the rebound hypertension that can be observed on cessation of a non-selective antagonist and was frequently reported in the BIRD-1 trial [15]. In the present study, there was no evidence that serum endothelin levels were increased on treatment, and no evidence of rebound or worsening hypertension occurring after cessation of zibotentan. This may make a selective ERA easier to use in the context of SSc-CKD and especially after SRC.

Since the original design of the ZEBRA trial, additional information is available regarding candidate biomarkers of SSc-CKD. It is apparent that whilst serum sVCAM-1 may be informative as a maker of SRC [18], it does not appear to reflect CKD in SSc. It is, therefore, not surprising that the primary end point for ZEBRA 1 was negative. However, other markers in an independent study of SSc-CKD were much more promising and in line with emerging data for other forms of connective tissue disease associated renal disease such as lupus nephritis [23], and some urinary proteins appear to be selectively increased in SSc-CKD. In general, urinary markers may be more informative in a multi-compartment disease than serum or blood analytes that could be affected by severity and activity of the disease process in multiple organs. Of the two candidate markers for SSc-CKD one showed some interesting changes in ZEBRA 1. Thus, for urinary MCP-1/creatinine ratio the baseline levels are comparable, but in patients receiving zibotentan there is a fall on zibotentan and a rise on placebo at 26 weeks with continued separation to 52 weeks. The number of treated patients in ZEBRA 1 is too small to reliably draw any conclusion but provides valuable data for this candidate marker of SSc-CKD that could inform future studies.

Although serum or urine protein analysis may give indirect insight into SSc-CKD progression or treatment, the data for renal function measurement by eGFR are more compelling. These are supportive of a potential treatment effect for zibotentan in ZEBRA 1, although the very small number of cases recruited means that all interpretation should be cautious, and no formal statistical comparison is possible. Nevertheless, as for urinary MCP1, there is a fall in eGFR at 26 and 52 weeks for the placebo-treated patients in ZEBRA 1, whereas on average there is numerical improvement at 26 weeks at the end of treatment and further clinically meaningful improvement only in those cases receiving zibotentan when assessed at the 52-week safety visit. This is notable because there is precedent for a treatment effect continuing after completion of therapy in a previous clinical trial of oral cyclophosphamide in SSc-associated lung fibrosis [24] and possibly also in the BIRD-1 trial of bosentan in SRC [14].

Our results for eGFR in the zibotentan group of ZEBRA 1 are in line with the findings for post SRC CKD in BIRD-1 [14], but more importantly they are aligned with recent high-quality data from a trial of another highly selective ERA in diabetes-associated CKD (Study of Diabetic Nephropathy with Atrasentan: SONAR [25]). Thus, the recently reported SONAR trial of atrasentan and renal events in patients with type 2 diabetes and chronic kidney disease (a double-blind, randomised, placebo-controlled trial, which randomised 2648 patients) demonstrated statistically significant and clinically meaningful benefit in slowing progression of CKD in type 2 diabetes [25]. The SONAR study used an innovative design that first identified responders to atrasentan in whom albumin to creatinine ratio improved by at least 30%, based on previous short-term studies [26] and a run-in period, and in whom there was not excessive fluid retention. These responders were subsequently randomised to placebo, or to continue ERA treatment. This approach also suggested longer term benefit in some cases that were randomised to placebo as the impact on albumin: creatinine ratio appeared blunted in the subsequent phase of the study suggesting longer term benefit from atrasentan received during the ‘responder determining’ phase. Despite this, the SONAR trial was strongly positive using a composite endpoint reflecting clinically meaningful CKD progression. These findings are highly supportive of the apparent improvement noted in eGFR at 52 weeks for the zibotentan group in ZEBRA 1.

Because it is predominantly renally excreted [27], there were concerns about using zibotentan in patients with impaired renal function and especially those on dialysis. This is important because in SRC, the major unmet need is to increase the proportion of patients requiring dialysis who can subsequently recover independent renal function. It has been shown previously that cases of SSc-CKD after SRC that need permanent dialysis have a very poor long-term outcome and survival [26]. This was the rationale for exploring zibotentan pharmacokinetics in patients on dialysis. The data provides valuable information to this end and suggests that intermittent dosing with 2.5 or 5 mg leads to therapeutic and non-toxic levels of zibotentan. The peak drug level in all subjects in ZEBRA 2 was less than half than those reported in a previous study examining 10mg doses in individuals with normal or impaired renal function [27], consistent with the lower dose used in our trial. Relatively low doses were used as this was the first study of zibotentan in haemodialysis. Peak plasma concentrations at these doses did not exceed those seen in previous studies in patients with either normal or impaired renal function [27]. Adequate clearance after 24 h (with further clearance observed across 4 hours of haemodialysis) implies daily dosing could be safely assessed in an extended study. These data are generalisable as ZEBRA 2B included patients with multiple underlying causes of renal failure, but in future could be of particular relevance to cases of SRC that require dialysis. These cases could be included in any future trial, and this would also be expected to facilitate recruitment especially in the important post-SRC population that was included in BIRD-1 and other recently reported studies [15].

These trials have several important limitations. The first is the small number of cases recruited to ZEBRA 1 and ZEBRA 2A. This precludes robust conclusions from our findings in any way that may immediately change clinical practice and limits interpretation to consideration of future additional trials. However, lessons learned undertaking these trials and the results related to pharmacokinetics and safety provide valuable information.

There are also some notable strengths. By focusing on SSc-CKD, it was possible to explore assessment and feasibility of trials in this patient group that has high unmet need. The testing of recently described candidate biomarkers of CKD, including urinary MCP-1 that has also proven informative in other studies of connective tissue disease-associated CKD, is of particular interest and may be explored further in future trials and clinical practice.

## Conclusions

Taken together, the ZEBRA clinical trial substudies represent an important step forward in better understanding and exploring SSc-CKD and provide support for the safety and potential efficacy of a highly selective endothelin receptor antagonist that can be explored in future trials. In addition, we have defined suitable dose and pharmacokinetics for zibotentan in patients on haemodialysis that may facilitate its use in other clinical trials and contexts.

## Data Availability

Data will be shared for purposes of academic research upon reasonable request.

## Abbreviations

SSc: Systemic sclerosis
SRC: Scleroderma renal crisis
CKD: Chronic kidney disease
eGFR: Estimated glomerular filtration rate
ESKD: End-stage kidney disease
KDIGO: Kidney Disease: Improving Global Outcomes
ET-1: Endothelin 1
ETRA: Endothelin receptor A
ETRB: Endothelin receptor B
MCP-1: Monocyte chemoattractant protein 1
sICAM-1: Soluble intercellular adhesion molecule 1
sVCAM-1: Soluble vascular cell adhesion molecule 1
ZEBRA: Zibotentan better outcome in renal scleroderma clinical trial
BIRD-1: Bosentan in renal disease clinical trial
CTD: Connective tissue disease
MDRD: Modification of Diet in Renal Disease Study
PK: Pharmacokinetic
LC-MS/MS: Liquid chromatography with tandem mass spectrometry
SONAR: Study of Diabetic Nephropathy with Atrasentan
